# Retinal structure and visual pathway function at school age in children born extremely preterm: a population-based study

**DOI:** 10.1186/s12886-023-03055-4

**Published:** 2023-07-01

**Authors:** Sigrid Hegna Ingvaldsen, Kyrre Moljord, Arnstein Grøtting, Petter Moe Omland, Olaf Dammann, Dordi Austeng, Tora Sund Morken

**Affiliations:** 1 Department of Neuromedicine and Movement Science, NTNU Norwegian University of Science and Technology, Trondheim, Norway; 2 Department of Ophthalmology, St. Olavs Hospital, Trondheim University Hospital, Trondheim, Norway; 3 Department of Neurology and Clinical Neurophysiology, St. Olavs Hospital, Trondheim University Hospital, Trondheim, Norway; 4 Department of Public Health and Community Medicine, Tufts University School of Medicine, Boston, MA USA; 5 Department of Gynecology and Obstetrics, Hannover Medical School, Hannover, Germany

**Keywords:** Optical coherence tomography, Visual evoked potentials, Extremely preterm, Retinopathy of prematurity

## Abstract

**Background:**

Children born extremely preterm (gestational age < 28 weeks) show reduced visual function even without any cerebral or ophthalmological neonatal diagnosis. In this study, we aimed to assess the retinal structure with optical coherence tomography (OCT) and visual function with pattern-reversal visual evoked potentials (PR-VEPs) in a geographically defined population-based cohort of school-aged children born extremely preterm. Moreover, we aimed to explore the association between measures of retinal structure and visual pathway function in this cohort.

**Methods:**

All children born extremely preterm from 2006–2011 (*n* = 65) in Central Norway were invited to participate. Thirty-six children (55%) with a median age of 13 years (range = 10–16) were examined with OCT, OCT-angiography (OCT-A), and PR-VEPs. The foveal avascular zone (FAZ) and circularity, central macular vascular density, and flow were measured on OCT-A images. Central retinal thickness, circumpapillary retinal nerve fibre layer (RNFL) and inner plexiform ganglion cell layer (IPGCL) thickness were measured on OCT images. The N70-P100 peak-to-peak amplitude and N70 and P100 latencies were assessed from PR-VEPs.

**Results:**

Participants displayed abnormal retinal structure and P100 latencies (≥ 2 SD) compared to reference populations. Moreover, there was a negative correlation between P100 latency in large checks and RNFL (*r* = -.54, *p* = .003) and IPGCL (*r* = -.41, *p* = .003) thickness. The FAZ was smaller (*p* = .003), macular vascular density (*p* = .006) and flow were higher (*p* = .004), and RNFL (*p* = .006) and IPGCL (*p* = .014) were thinner in participants with ROP (*n* = 7).

**Conclusion:**

Children born extremely preterm without preterm brain injury sequelae have signs of persistent immaturity of retinal vasculature and neuroretinal layers. Thinner neuroretinal layers are associated with delayed P100 latency, prompting further exploration of the visual pathway development in preterms.

**Supplementary Information:**

The online version contains supplementary material available at 10.1186/s12886-023-03055-4.

## Introduction

Children born extremely preterm (gestational age (GA) < 28 weeks) are at increased risk of visual impairments [1] that are not fully explained by sequelae from retinopathy of prematurity (ROP) [2]. The disease is defined by clinically observed pathological neovascularisation of the retina during development and retinal detachment in its end stage, resulting in visual impairment [3]. However, neonatal screening identifies individuals needing treatment. Therefore, end-stage ROP is rarely seen in high-income countries [4] and may thus not be the primary contributor to the increased risk of visual impairments observed in children born extremely preterm. Moreover, it has been suggested that ROP is not only a vascular disease but also includes injury to the neurovascular interphase in the retina and/or brain [5]. Indeed, we have suggested that ROP is merely the tip of the iceberg of a more extensive entity coined "Visuopathy of Prematurity" (VOP), which is proposed to encompass neurovascular tissue injury in the retina and the cerebral visual pathways of children born preterm [2].

The fovea is not fully matured until one or two years of age because most inner retinal differentiation occurs before birth, and outer retinal differentiation occurs after birth [6]. During foetal development, the fovea is formed by the centrifugal movement of inner retinal layers to the periphery and migration of the outer photoreceptor layers towards the centre of the foveola [7, 8]. The vascular mesh that covers the retina in early foetal life retracts to leave an avascular zone in the fovea, facilitating light to access foveolar photoreceptors and enabling sharp vision [9]. This process occurs during late gestation, and both the development of the retinal vasculature and the neuroretina may be interrupted by factors associated with preterm birth.

Optical coherence tomography (OCT) angiography provides non-invasive in vivo high-resolution imaging of the retinal layers and vasculature and has revealed retinal microstructural abnormalities such as a smaller foveal avascular zone, higher vascular density, and a thicker central macula among children born preterm at age 3–17 years old [10–12]. The retinal abnormalities might also be associated with abnormal functional integrity of the visual pathways. Indeed, altered visual evoked potentials (VEPs) generated in the occipital cortex have been reported in very low birth weight (VLBW; < 1500 g birthweight) pre-schoolers [13]. However, the association between OCT and VEP measures has not yet been explored in school-aged children born extremely preterm. Moreover, research findings are inconsistent on whether the pattern of abnormal retinal structure and visual function is more prominent among children born extremely preterm with ROP [14, 15].

Thus, we wanted to assess the structure of the retinal neurovascular development, the visual pathways' function, and possible associations in a population-based cohort of school-aged children born extremely preterm with and without ROP.

## Methods and materials

### Study design and participants

All children residing in Norway who were born extremely preterm from 2006–2011 in the geographical region of Central Norway were identified via the Norwegian Neonatal Network (NNK), a national medical quality registry that collects data on all new-borns admitted to neonatal intensive care units in Norway. Norway has near-universal health insurance coverage. Information from NNK was cross-checked with medical records, and the children's last names were cross-checked with the Norwegian National Population Register to obtain the addresses of their parents. The study had no exclusion criteria. Sixty-five children were invited via a mailed letter containing information about the study. Parents were contacted by phone for consent; 14 could not be reached. Of the remaining 51, 36 (55%) consented and were enrolled in the study between March 3 and September 2, 2021. Their median (range) age at visual assessment was 13 years (10–16). Background neonatal data were obtained from NNK and cross-checked in medical records for participants and non-participants (those who declined to participate and could not be reached). There were no significant differences between participants and non-participants in clinical background data [16]. A clinical assessment of brain MRI had been performed as part of an earlier study and confirmed that none of the participants had signs of preterm brain injury [16].

### Ophthalmological examination

Best-corrected visual acuity (BCVA) as letter score and the logarithm of the minimum angle of resolution (logMAR) was obtained monocularly and binocularly following subjective refraction at 4 m distance according to the Early Treatment Diabetic Retinopathy Study (ETDRS) [17]. Subsequent testing was performed with the best correction. Cut-off scores for BCVA were calculated as < 85 letter score (equivalent to 20/20 Snellen and a logMAR score of 0.0), which is clinically regarded as normal vision.

### Optical coherence tomography

After dilation with one drop of phenylephrine 10% and cyclopentolate 1%, OCT and OCT angiography (OCT-A) were obtained using the Zeiss Cirrus 6000 (Carl Zeiss Meditec, Inc., California, USA). We obtained 512 × 128 mm images from the macula, 200 × 200 mm images from the optic disc, and 3 × 3 mm OCT-A images for both eyes.

**Fig. 1:**
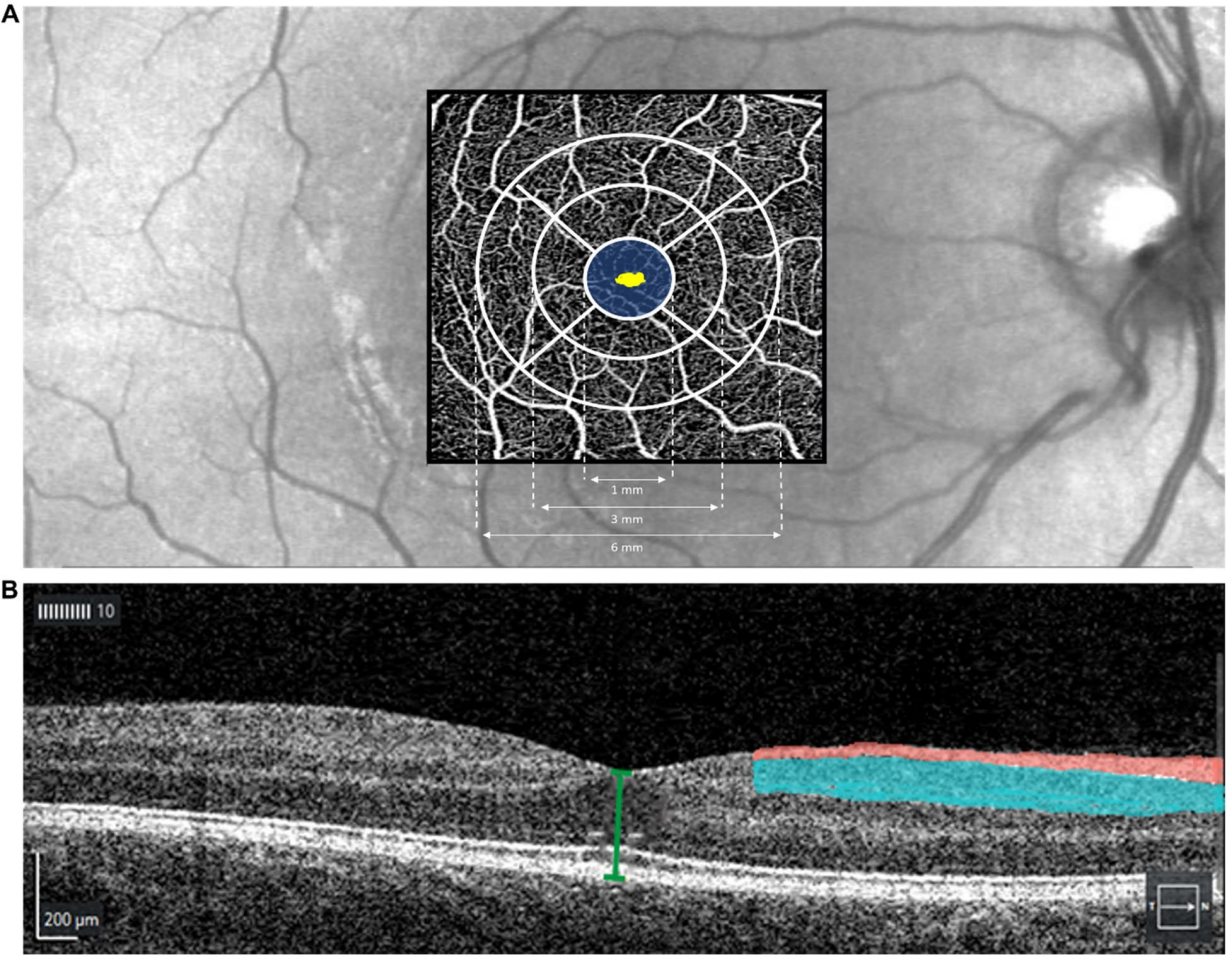
Illustration of the OCT parameters extracted for analysis. **A** OCT-A image with the foveal avascular zone (yellow area) and the central macular thickness measured in 1 diameter (blue area). **B** OCT B-scan image of the retina with the central retinal thickness (green line) measured from the inner limiting membrane to the retinal pigment epithelium, the retinal nerve fibre layer thickness (red area), and the inner plexiform ganglion cell layer (blue area)

Macular vascular density (MVD; mm/mm^2^) in the superficial central area, macular vascular flow (MVF; %) in the superficial central area, foveal avascular zone (FAZ) area (mm^2^), and FAZ circularity were obtained from OCT-A images using the AngioPlex Metrix software. In addition, circumpapillary retinal nerve fibre layer (RNFL) thickness (µm) and macular inner plexiform ganglion cell layer (IPGCL) thickness (µm) were automatically quantified and obtained from OCT images (Fig. 1).

**Fig. 2:**
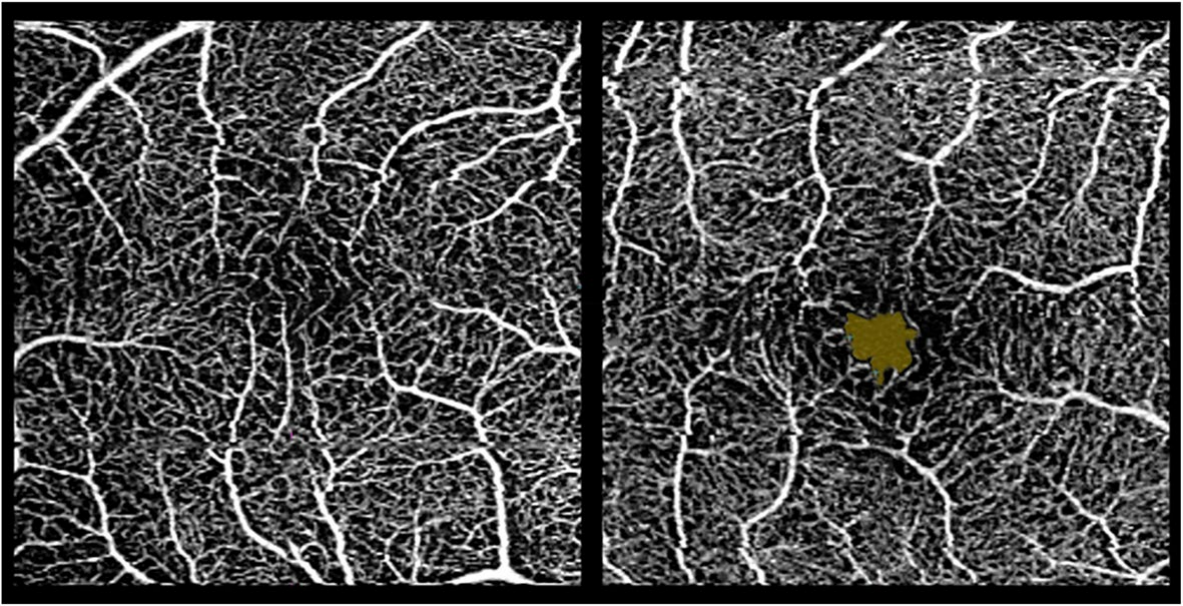
OCT-A images illustrating the FAZ area (yellow area) of a participant with (right) and without (left) ROP. FAZ = foveal avascular zone; OCT = optical coherence tomography; ROP = retinopathy of prematurity. The FAZ area is not visible in the participants with ROP due to blood vessels going through the fovea

For eyes in which blood vessels crossed the fovea, the FAZ area was set to zero (Fig. 2). Mean central macular thickness (CMT; µm) in the fovea was obtained using the macular cube 512 × 128 protocol, where macular thickness data are presented in nine ETDRS areas. The central subfield (A1) measures 1 mm in diameter and was used to calculate CMT. Central retinal thickness (CRT; µm) was measured manually from the inner limiting membrane to the retinal pigment epithelium (Fig. 1).

Cut-off scores defined as the limit for abnormal values for the FAZ area (< 0.3 mm/mm^2^) and FAZ circularity (< 0.7) were calculated based on reference values obtained from a PlexElite 9000 (Carl Zeiss Meditec, Inc., California, USA) OCT machine from a group of 19 children (6–8 years old at examination) born at term [18]. Cut-off scores for RNFL thickness (< 83 µm) were based on reference values obtained on a spectral domain Cirrus (version 6.0.2.81, Carl Zeiss Meditec, Inc., California, USA) from a group of 57 children (6–15 years old at examination) born at term [19]. Cut-off scores for IPGCL thickness (< 99 µm) were based on reference values obtained on a Cirrus HD-OCT (Carl Zeiss Meditec, Inc., California, USA) from a group of 114 children (mean age 8.1 years old at examination) born at term [20]. Cut-off scores for central macular thickness (> 255 µm) were calculated based on reference values measured with spectral-domain Cirrus (version 6.0.2.81, Carl Zeiss Meditec, Inc., California, USA) from a group of 57 children (6–15 years old at examination) born at term [21].

### Visual evoked potentials

Pattern-reversal visual evoked potentials (PR-VEPs) from the left and right eye were recorded on a Keypoint computer (Keypoint, Neurolite Software, Natus, Switzerland) using a View Sonic Graphics Series 670 fmb CRT monitor (17 inches). Electrodes were placed according to the 10–20 system on occipital, frontal, and parietal areas (Oz, Fz, and Pz) [22]. PR-VEPs were recorded from the occipital midline (Oz) and referred to the mid-frontal electrode (Fz) according to the ISCEV standards [23]. Impedance was < 5 kΩ, and a 1 Hz-1 kHz filter was used. The rejection level was set to ± 90 µV.

The subjects were seated in a relaxed position in a chair with neck support. One eye was covered with an eyepatch. The PR-VEP task consisted of high-contrast black-and-white checks with a red fixation point in the middle of the checkboard. The PR-VEP recording was performed in a dark room at 1 m from the CTR monitor. PR-VEPs were recorded in one eye at a time with 66' (12 × 16) (large checks) and 16' (48 × 64) (small checks) checks with 100 stimulations per run and a stimulations frequency of 1 Hz. A minimum of two runs for each eye and check size were conducted to assess the reproducibility of the responses. Two reproducible responses were required to be reliable for analysis.

An experienced specialist in clinical neurophysiology (AG) blinded for ROP status visually identified and placed cursors on N70, P100, and N145 peaks. The N70 and P100 latency (ms) and peak-to-peak N70-P100 amplitude (μV) were obtained for analysis. Latencies were measured from stimulus onset to the peak of the N70 and P100 waves, and amplitude was measured between the N70 and P100 peaks. Cut-off scores for PR-VEP potentials were calculated as ≥ 113.5 ms (+ 2 SD) and ≥ 119.3 ms (+ 3 SD) for the P100 latency of small checks, and ≥ 109.4 ms (+ 2 SD) and ≥ 114.8 ms (+ 3 SD) for P100 latency of large checks. The cut-off for the VEP amplitude was calculated as ≤ 3.9 µV (-2 SD) and ≤ 2.5 µV (-3 SD) for the small checks and ≤ 2.7 µV (-2 SD) and ≤ 1.5 (-3 SD) for the large checks. The cut-off scores are based on local reference values collected from an adult population (*n* = 93 and *n* = 96 for small and large checks, respectively) at the Department of Clinical Neurophysiology at St. Olavs Hospital, Trondheim, Norway.

### Statistical analyses

All statistical analyses were conducted using the SPSS software 27.0 (IBM, New York, USA) and RStudio 4.2 (PBC, Boston, MA). Histograms and Q-Q plots were visually inspected to assess the normality of the data distributions. The FAZ area, FAZ circularity, and N70 latency showed non-normally distributions. However, due to the small sample size, we used parametric tests for all analyses to reduce the likelihood of Type II errors. All analyses were performed using data from the eye with the best corrected visual acuity (better eye). If the BCVA ETDRS letter score were equal in both eyes, the right eye was chosen for analyses.

Independent two-sample t-tests with *p*-values adjusted for unequal variances were performed to assess differences in scores on OCT parameters and PR-VEP variables between participants with and without ROP. Further, a partial Pearson's correlation analysis controlling for the effect of age was performed to investigate the relationship between PR-VEP variables and ganglion cell layer thickness. In addition, the relationship between gestational age and study variables (OCT parameters and PR-VEP variables) and the relationship between best corrected visual acuity and OCT parameters were explored with Pearson´s correlation analysis.

One participant had missing OCT data due to excessive movement during the examination, which caused unclear images. Two participants had missing VEP data points due to 50 Hz noise making it difficult to identify the VEP components. In addition, two participants had missing VEP and OCT data due to nystagmus, and P100 latency for one subject with large checks was excluded because the component could not reliably be identified.

## Results

### Ophthalmological examination

The standardised medical ocular history has been published earlier [16]. In brief, two participants had nystagmus (6%), five (14%) had been treated for amblyopia, and 15 (42%) used glasses or lenses. Mean intraocular pressure was 17.5 (SD = 3.6) mmHg for the better eye and 17.0 (SD = 3.5) for the worse eye (normal range of IOP 6–21 mmHg).

Twenty-six participants had no history of ROP, while 7 participants (19%) had a history of ROP. Of the children with ROP, two developed Type I and were treated. One participant had Type II that regressed, and four had mild ROP (stage 2). The mean BCVA ETDRS letter score for the better eye was 74.6 (SD = 18.8) for participants with ROP and 86.0 for participants without ROP (SD = 4.1), and almost half (49%) of the participants had an ETDRS letter score lower than 85 (equivalent to Snellen 20/20 and logMAR 0.0) in their better eye [16].

### Retinal structure (OCT)

Most participants displayed an abnormal retinal structure (Table 1). Almost all participants (97%) had a smaller FAZ area (mean = 0.05, SD = 0.08), and 93% had a poorer FAZ circularity than the cut-off (mean = 0.30, SD = 0.30). In addition, 77% displayed a thinner RNFL (mean = 91.8, SD = 10.1), and 60% had a thinner IPGCL than the cut-off (mean = 81.8, *SD* = 6.7). The central macular thickness was thicker than the cut-off in all participants (mean = 291.4 µm, SD = 19.3). Central retinal thickness was 274.2 µm (SD = 26), mean macular vascular density was 17.2 mm/mm^2^ (SD = 3.0), and mean macular vascular flow was 32% (SD = 4.8). Results from the correlation analysis between best corrected visual acuity and OCT parameters are presented in Additional file 1. A small positive correlation was found between best corrected visual acuity and central macular thickness, indicating a thicker central macula with better visual acuity in the participants (*r* = 0.27, *p* = 0.126).

**Table 1:** OCT and PR-VEP variables for the better eye in participants presented by gestational age

Gestational age (weeks)				
	≤ 24	**25**	**26**	**27**
	(*n* = 6)	(*n* = 5)	(*n* = 7)	(*n* = 15)
**OCT parameters**				
FAZ (mm^2^)	.03 ± .04^a^	.03 ± .04^a^	.01 ± .02^a^	.07 ± .11^a^
< *0.3 mm*^*2*^* (n (%))*	5 (100)	4 (100)	6 (100)	13 (93)
FAZ circularity	.20 ± .30^a^	.30 ± .30^a^	.20 ± .30^a^	.30 ± .30^a^
< *0.7 (n (%))*	5 (100)	4 (100)	6 (100)	12 (86)
MVD (mm/mm^2^)	18.0 ± 1.9^a^	18.3 ± 3.3	17.8 ± 1.3^a^	16.2 ± 3.7^a^
MVF (%)	33.0 ± 3.4^a^	31.8 ± 4.0	32.5 ± 1.8^a^	31.0 ± 6.3^a^
CMT (µm)	299.2 ± 27.4	285.8 ± 11.0	297.0 ± 7.5	287.6 ± 21.4
> *255 µm (n (%))*	6 (100)	5 (100)	7 (100)	15 (100)
CRT (µm)	283.3 ± 25.8	274 ± 15.2	288.6 ± 21.2	264 ± 28.2
RNFL thickness (µm)	88.0 ± 10.8^a^	92.6 ± 10.1	87.2 ± 12.2^a^	94.9 ± 8.7^a^
< *99 µm (n (%))*	4 (80)	4 (80)	5 (83)	10 (71)
IPGCL thickness (µm)	80.4 ± 4.0^a^	81.8 ± 10.1	75.7 ± 5.7^a^	84.9 ± 4.7^a^
< *83 µm (n (%))*	4 (80)	3 (60)	6 (100)	5 (36)
**PR-VEP variables**				
(66’) N70 latency (ms)	68.0 ± 3.3^a^	66.0 ± 2.3^a^	72.3 ± 13.8	65.7 ± 5.6
(66’) P100 latency (ms)	107.4 ± 9.3^a^	103.9 ± 8.6^a^	110.0 ± 19.3	101.0 ± 5.1^a^
≥ *109.4 ms (*+ *2 SD) (n (%))*	1 (20)	1 (25)	3 (43)	1 (7)
≥ *114.8 ms (*+ *3 SD) (n (%))*	1 (20)	1 (25)	2 (29)	0
(66’) N70-P100 µV	19.8 ± 8.1^a^	17.5 ± 9.2^a^	15.8 ± 7.8	19.5 ± 10.3^a^
(16’) N70 latency (ms)	75.8 ± 2.0^a^	76.2 ± 3.9^a^	81.4 ± 10.3	74.3 ± 6.2^a^
(16’) P100 latency (ms)	120.2 ± 13.2^a^	115.5 ± 6.2^a^	109.3 ± 13.6	109.8 ± 8.9^b^
≥ *113.5 ms (*+ *2 SD) (n (%))*	3 (60)	3 (75)	2 (29)	5 (36)
≥ *119.3 ms (*+ *3 SD) (n (%))*	2 (40)	1 (25)	1 (14)	2 (14)
(16’) N70-P100 µV	18.7 ± 11.3^a^	13.7 ± 9.3^a^	13.9 ± 7.2	18.2 ± 8.7^a^

Data are presented as mean ± SD with n (%) of the participants below/above cut-off scores (described in the methods section), presented by gestational age at birth

*BCVA* best corrected visual acuity, *CMT* central macular thickness, *CRT* central retinal thickness, *FAZ* foveal avascular zone, *IPGCL* inner plexiform layer, *mm*^*2*^ square millimetre, *ms* milliseconds, *MVD* macular vascular density, *MVF* macular vascular flow, *RNFL* retinal nerve fibre layer, *µV* amplitude, *µm* micrometre, *66’* large checks, *16’* small checks

^a^Data are missing for one participant

^b^Data are missing for two participants

Participants without ROP had a thinner central retina than those with ROP (*p* = 0.032). In addition, participants with ROP displayed thinner RNFL (*p* = 0.006) and IPGCL thickness (*p* = 0.014), a higher macular vascular density (*p* = 0.006), and macular vascular flow (*p* = 0.004) compared to participants without ROP (Table 2). Furthermore, none of the participants with ROP had a measurable FAZ area, while this was the case for 5 of 29 participants (17%) in the no-ROP group (Fig. 2).

**Table 2:** OCT and PR-VEP variables for the better eye between participants with and without ROP

	**ROP (*n*= 7)**	**No-ROP (*n*= 33)**		
	**Mean ± SD**	**Mean ± SD**	***p*-value**	**95% CI**
**OCT parameters**				
FAZ (mm^2^)_1_	.00 ± .00_**6**_	.06 ± .09	.003	(.02, .09)
FAZ circularity_1_	.00 ± .00_**6**_	.03 ± .03	.000	(.03, .04)
MVD (mm/mm^2^)_2_	19.8 ± 1.6	16.7 ± 3.0	.006	(-5.1, -1.1)
MVF (%)_2_	34.6 ± 1.1	31.1 ± 5.0	.004	(-5.7, -1.2)
CMT (µm)_3_	297.0 ± 23.6	290 ± 18.3	.515	(-5.1, 1.1)
CRT (µm)_3_	295.7 ± 23.6	268.5 ± 23.3	.032	(-51.6, -2.9)
RNFL thickness (µm)_2_	80.4 ± 7.0	94.1 ± 9.0	.006	(5.2, 22.2)
IPGCL thickness (µm)_2_	74.6 ± 5.1	83.2 ± 6.0	.014	(2.4, 14.9)
**PR-VEP variables**				
(66’) N70 latency (ms)_4_	75.3 ± 15.6	66.1 ± 4.6	.258	(-28.6, 10.1)
(66’) P100 latency (ms)_2_	117.1 ± 19.7	102.0 ± 6.6	.163	(-39.4, 9.2)
(66’) N70-P100 µV_4_	16.6 ± 9.7	18.8 ± 9.0	.645	(9.5, 14.0)
(16’) N70 latency (ms)_2_	82.7 ± 12.3	75.2 ± 5.0	.248	(-22.7, 7.7)
(16’) P100 latency (ms)_5_	123.0 ± 16.6	110.0 ± 8.3	.156	(-33.3, 7.3)
(16’) N70-P100 µV_2_	13.5 ± 8.0	17.3 ± 8.9	.377	(-5.9, 13.6)

Mean ± SD and *p*-value with 95% CI from independent two-sample t-tests corrected for unequal variances for OCT and PR-VEP variables with ROP status as the grouping variable

*BCVA* best corrected visual acuity, *CMT* central macular thickness, *CRT* central retinal thickness, *FAZ* foveal avascular zone, *IPGCL* inner plexiform layer, *mm*^*2*^ square millimetre, *ms* milliseconds, *MVD* macular vascular density, *MVF* macular vascular flow, *RNFL* retinal nerve fibre layer, *uV* amplitude, *µm* micrometre, *66’* large checks, *16’* small checks

_1_ROP (*n*= 4), No-ROP (*n*= 25); _2_ROP (*n*= 5), No-ROP (*n*= 25); _3_ROP (*n*= 7), No-ROP (*n*= 26); _4_ROP (*n*= 5), No-ROP (*n*= 26); _5_ROP (*n*= 5), No-ROP (*n*= 24)

_6_The foveal avascular zone was not measurable in participants with ROP

### Visual pathway function (PR-VEPs)

were born before 26 gestational weeks. Sixlarge checks66') the reference values of + 2 SD cut-off (Table 2). Also, one participant born at 26 weeks GA displayed a weaker N70-P100 amplitude than the reference population. Mean P100 latencies (small checks/large checks) was 123/117.1 ms for those with ROP compared with 110/102 ms for those without ROP (Table 1). The correlation analysis between gestational age and study variables (Additional file 2) indicated longer P100 latencies with lower gestational ages in small checks (*r* = -0.34, *p* = 0.074) and large checks (*r* = -0.36, *p* = 0.090).

### Associations between ganglion cell layer thickness and PR-VEP variables

**Fig. 3:**
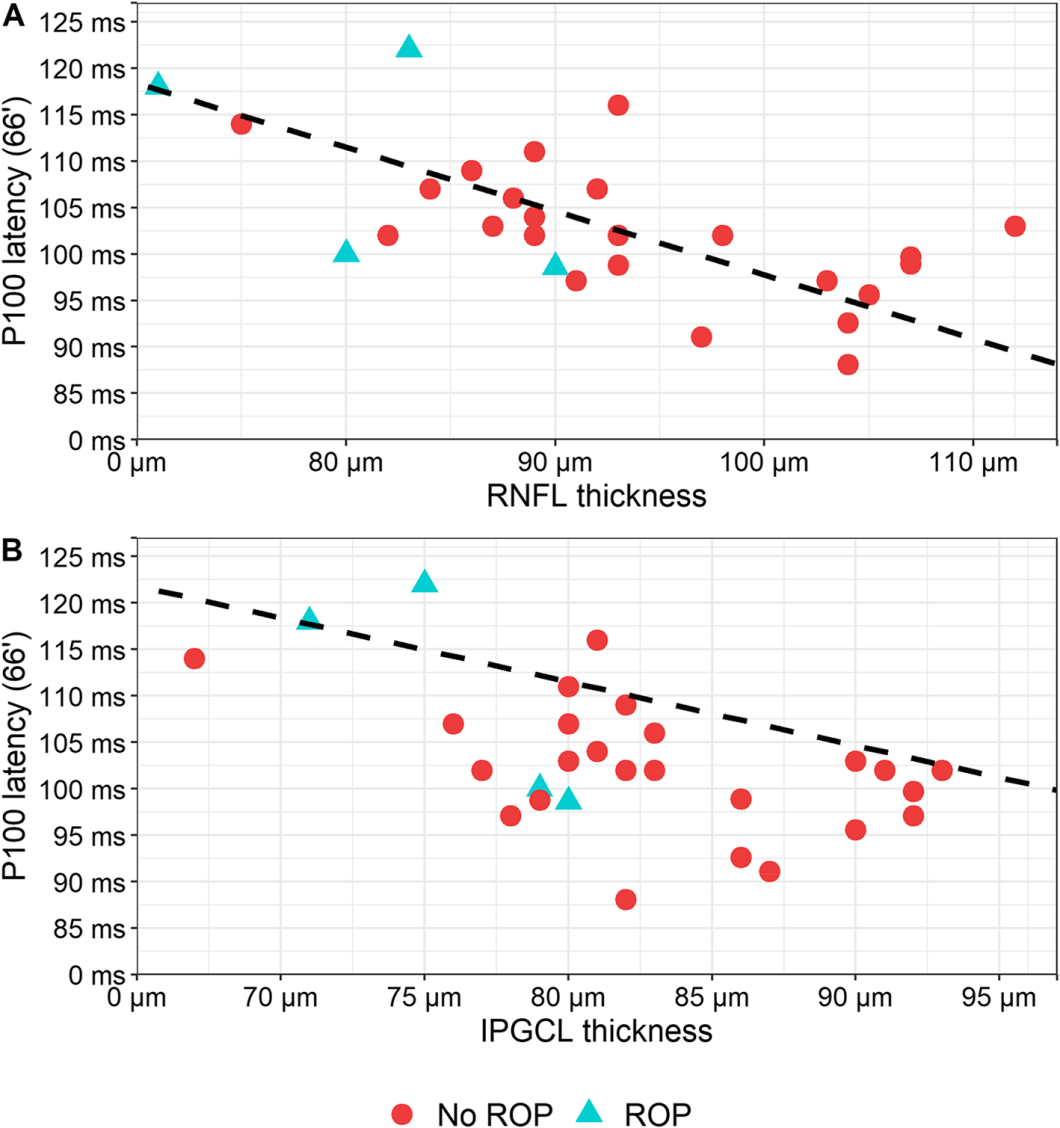
Association between P100 latency and ganglion cell layers thickness in participants with and without ROP. IPGCL = inner plexiform ganglion cell layer; ms = milliseconds; r = Pearson’s correlation coefficient; RNFL = retinal nerve fibre layer; ROP = retinopathy of prematurity. The association between RNFL thickness (**A**), IPGCL thickness (**B**) (x-axis) and P100 latency (y-axis) with Pearson’s correlation coefficient and Deming regression line for two dependent variables measured with error (dashed line). Blue triangles represent participants with ROP (*n* = 4), while red circles represent participants without ROP (*n* = 24)

Both IPGCL and RNFL showed a significant negative correlation with P100 latency (large checks; 66') (IPGCL: *r* = -0.53, *p* = 0.005; RNFL: *r* = -0.64, *p* < 0.001). Figure 3 presents the P100 latency (66') data distribution and IPGCL and RNFL thickness.

## Discussion

In a geographically defined population of school-aged children born extremely preterm, thinner neuroretinal layers were associated with delayed P100 latencies. Also, several participants displayed delayed P100 latencies, and all children born extremely preterm had abnormal central macula thickness compared to reference populations. In addition, individuals with ROP showed signs of immature foveal vasculature and neuroretinal layers.

To the best of our knowledge, the association between neuroretinal layers and P100 latencies in large checks in a cohort of school-aged children born extremely preterm without identifiable cerebral abnormalities has not been reported earlier. However, a similar association has been observed in patients with multiple sclerosis and optic neuritis [24, 25]. The two major parallel visual pathways, the magnocellular and parvocellular pathways, begin in the retina and project to the primary visual cortex via the lateral geniculate nucleus. The parvocellular pathway responds to high spatial resolution (small checks), low luminance contrast sensitivity, and low temporal resolution. In contrast, the magnocellular pathway responds to low spatial resolution (large checks), high luminance contrast sensitivity, and high temporal resolution [26–28]. The association between delayed P100 latencies in large checks with thinner ganglion cell layers in this study may indicate an abnormal development of the magnocellular pathway, activated in low spatial frequency stimulus conditions. Moreover, several participants had abnormal P100 latencies with small checks (16'), indicating a delayed development of the parvocellular pathways in school-aged children born extremely preterm.

While 39% of our participants had delayed P100 latencies compared to norms in the small checks, only 18% had delayed P100 latencies in the large checks. A previous study found that children (with a mean age of 5.8 years) born late preterm (GA week 32–37) exhibited no significant differences in P100 latency compared to children born at term [29], while another study found that children (4–6 years of age) born with GA between 28 and 32 weeks had significantly delayed P100 latencies compared to controls [13]. These conflicting results may be due to the difference in gestational age. Indeed, correlations analysis indicated lower gestational age with delayed P100 latencies in this study.

The findings of delayed P100 latencies in higher spatial frequencies (smaller checks) are consistent with our earlier findings that approximately 60% of participants from the same study sample had lower than normal contrast sensitivity abilities, especially in higher spatial frequencies [16] which might indicate a delayed development of the parvocellular pathway. The parvocellular pathway's high-resolution capacity in the fovea results from the close synaptic connectivity between cones and projecting bipolar and ganglion cells [30]. Cone photoreceptor anomalies caused by abnormal retinal development could explain the delayed latencies from small checks conveyed by the parvocellular pathway. Indeed, decreased cone-mediated pupillary response to photopic stimuli has been shown in children born preterm with macular developmental arrest characterised by a shallowed pit with significantly reduced outer nuclear layer to inner retinal layer ratio in the fovea [31, 32]. Cone anomalies in the fovea due to an immature retina at birth could cause poor synaptic connections with projecting ganglion cells, leading to slower VEPs reaching the visual cortex for processing.

Our findings of more prominent foveal anomalies in participants with ROP align well with earlier findings of a decreased FAZ area and circularity in 6–13-year-old children born preterm with ROP [11, 14, 18]. The FAZ area mainly comprises elongated photoreceptors, and its lack of vascularity facilitates sharp vision in the foveola. When the development of the retinal vasculature is interrupted by, for instance, preterm birth, it can affect several aspects of vision. A small FAZ area with capillaries and astrocytes close to the fovea may disrupt the inner retinal layers' centrifugal migration during development, leading to abnormal visual development [33]. In our study, approximately half of the participants without ROP had no measurable FAZ area, while this was true for all participants with ROP, suggesting that factors associated with preterm birth are also risk factors for abnormal FAZ development in general but more prominent in those born preterm with ROP. The increased central macular thickness observed in this study cohort corroborates previous findings of increased central macular thickness in preterm children compared to children born to term with similar ages [7, 14, 34]. In this study, the ROP status did not have a large impact on central macular thickness. However, the small number of participants with ROP means that the results should be interpreted cautiously.

This study found a small positive correlation between best corrected visual acuity and central macular thickness. Few studies have assessed the relationship between visual acuity and retinal structure in extremely preterm children, and those who have report inconsistent findings [9]. One study assessing children born preterm between 5–16 years of age found no significant correlation between macular thickness and visual acuity, which coincides with our study [7]. However, one study found that inner retinal thickness was associated with suboptimal visual acuity in children (5–8 years) born preterm with a history of treated ROP [12]. The inconsistencies in findings could be due to the different exclusion criteria of patients with reduced visual acuity due to movement artefacts (from poor fixation/nystagmus) affecting the OCT image quality. The small correlation found in this study indicated that the relationship between visual acuity and retinal structure should be studied in larger samples.

The findings of this paper indicate that school-aged children born extremely preterm have an abnormal neuroretinal structure associated with delayed visual pathway signalling in the visual axis of the central nervous system. Furthermore, there were signs of abnormal vascular development even in the absence of ROP. This may indicate that visual function in individuals born preterm may be altered due to processes at the retinal as well as the cerebral level of the visual system. The retina is an extension of the central nervous system, and it is conceivable that normal neurovascular development may be interrupted by preterm birth at both sites. For instance, it has been observed that brain abnormalities in children with ROP are frequent [5]. However, there is a lack of studies prospectively designed to answer whether ROP and brain abnormalities following preterm birth may be part of the same pathogenesis with a common root cause in neurovascular development.

### Strengths and limitations

A strength of this study is the geographically defined population-based research design, inviting all children born extremely preterm within a geographically defined area during a specific time period. Moreover, the similarity of neonatal background data from participants and non-participants suggests that our findings are representative of the larger population of children born extremely preterm in Norway. A limitation of this study is its small sample size, especially the low number of participants with ROP. Although some differences between those with and without ROP did not achieve formal statistical significance, probably at least in part due to insufficient statistical power, the observed differences in retinal maturity and PR-VEP latencies were large and could still be of clinical significance. The comparisons between individuals with and without ROP should therefore be investigated in larger populations. We applied cut-offs based on normative data obtained locally as recommended by the International Federation of Clinical Neurophysiology [35] and the International Society for Clinical Neurophysiology of Vision [23]. The reference VEP data were from an adult and not a paediatric population. Although P100 latency attains adult-like values from 5 years of age [36], P100 latency shows some decline from 5 to 19 years of age [37]. This could lead to slightly overestimating the number of abnormal P100 values.

## Conclusion

In a geographically defined population of school-aged children born extremely preterm without preterm brain injury, ganglion cell layer thickness and retinal nerve fibre layer thickness were negatively correlated with P100 latencies, indicating a relationship between the neuroretina and visual pathway function among children born extremely preterm. Furthermore, most participants displayed an immature structure of the neuroretina and vasculature. ROP status was associated with differences in OCT(-A) parameters but not visual evoked potentials. Our results suggest that OCT(-A) and PR-VEP findings might be markers for VOP and should be studied in larger populations of children born extremely preterm to determine their clinical usefulness for the identification of visual pathway abnormalities in preterm children.

## Supplementary Information

**Additional file 1.** Correlation table showing the correlation coefficients and *p*-value for the associations of best corrected visual acuity with OCT parameters.

**Additional file 2.** Correlation table showing the correlation coefficients and *p*-value for the associations of gestational age with OCT parameters and PR-VEP variables.

## Data Availability

The datasets used and/or analysed during the current study are available from the corresponding author on reasonable request.

## Abbreviations

BCVA: Best corrected visual acuity
CMT: Central macular thickness
CRT: Central retinal thickness
ETDRS: Early Treatment Diabetic Retinopathy Study
FAZ: Foveal avascular zone
GA: Gestational age
IPGCL: Inner plexiform ganglion cell layer
logMar: Logarithm of the minimum angle of resolution
MRI: Magnetic resonance imaging
MVD: Macular vascular density
MVF: Macular vascular flow
NNK: Norwegian Neonatal Network
OCT: Optical coherence tomography
OCT-A: Optical coherence tomography angiography
PR-VEPs: Pattern-reversed visual evoked potentials
RNFL: Retinal nerve fibre layer
ROP: Retinopathy of prematurity
SD: Standard deviation
VEPs: Visual evoked potentials
VLBW: Very low birth weight
VOP: Visuopathy of Prematurity
