# Supplementary material for: Retinal structure and visual pathway function at school age in children born extremely preterm: a population-based study
Source: BMC Ophthalmol. 2023 Jul 1;23:296. doi: 10.1186/s12886-023-03055-4 (PMC10315021; doi:10.1186/s12886-023-03055-4)
Supplement: Supplementary file 1 — Additional file 1. Correlation table showing the correlation coefficients and p-value for the associations of best corrected visual acuity with OCT parameters. [file 12886_2023_3055_MOESM1_ESM.docx]

**Table A.** Correlation analysis of best corrected visual acuity and OCT parameters

|  | **Best corrected visual acuity** | |
| --- | --- | --- |
|  | Correlation coefficient (r) | p-value |
| **OCT parameters** | | |
| FAZ (mm^2^) **^a^** | -.07 | .718 |
| FAZ circularity **^a^** | .02 | .932 |
| MVD (mm/mm^2^) **^b^** | .08 | .690 |
| MVF (%) **^b^** | .20 | .291 |
| CMT (µm) | .27 | .126 |
| CRT (µm) | .05 | .773 |
| RNFL thickness (µm) **^b^** | .08 | .660 |
| IPGCL thickness (µm) **^b^** | -.13 | .502 |

CMT= central macular thickness; CRT= central retinal thickness; FAZ= foveal avascular zone; IPGCL= inner plexiform layer; mm^2^= square millimetre; MVD= macular vascular density; MVF= macular vascular flow; RNFL= retinal nerve fibre layer.

**^a^** Data are missing for four participants

**^b^** Data are missing for three participants
