# Supplementary material for: Retinal structure and visual pathway function at school age in children born extremely preterm: a population-based study
Source: BMC Ophthalmol. 2023 Jul 1;23:296. doi: 10.1186/s12886-023-03055-4 (PMC10315021; doi:10.1186/s12886-023-03055-4)
Supplement: Supplementary file 2 — Additional file 2. Correlation table showing the correlation coefficients and p-value for the associations of gestational age with OCT parameters and PR-VEP variables. [file 12886_2023_3055_MOESM2_ESM.docx]

**Table B.** Correlation analysis of gestational age with study variables

|  | **Gestational age** | |
| --- | --- | --- |
|  | Correlation coefficient (r) | p-value |
| **OCT parameters** | | |
| FAZ (mm^2^) **^a^** | .17 | .389 |
| FAZ circularity **^a^** | .06 | .747 |
| MVD (mm/mm^2^) **^b^** | -.23 | .224 |
| MVF (%) **^b^** | -.12 | .545 |
| CMT (µm) | -.19 | .297 |
| CRT (µm) | -.23 | .193 |
| RNFL thickness (µm) **^b^** | .20 | .291 |
| IPGCL thickness (µm) **^b^** | .26 | .162 |
| **PR-VEP variables** | | |
| (66’) N70 latency (ms) **^c^** | -.07 | .965 |
| (66’) P100 latency (ms) **^b^** | -.36 | .090 |
| (66’) N70-P100 µV **^c^** | -.08 | .699 |
| (16’) N70 latency (ms) **^c^** | -.01 | .709 |
| (16’) P100 latency (ms) **^b^** | -.34 | .074 |
| (16’) N70-P100 µV **^c^** | -.08 | .699 |

CMT= central macular thickness; CRT= central retinal thickness; FAZ= foveal avascular zone; IPGCL= inner plexiform layer; mm^2^= square millimetre; ms= milliseconds; MVD= macular vascular density; MVF= macular vascular flow; RNFL= retinal nerve fibre layer; uV= amplitude; µm= micrometre; 66’= large checks; 16’= small checks.

**^a^** Data are missing for four participants

**^b^** Data are missing for three participants

**^c^** Data are missing for two participants
